# The arginine metabolome in acute lymphoblastic leukemia can be targeted by the pegylated‐recombinant arginase I BCT‐100

**DOI:** 10.1002/ijc.31170

**Published:** 2017-12-26

**Authors:** Carmela De Santo, Sarah Booth, Ashley Vardon, Antony Cousins, Vanessa Tubb, Tracey Perry, Boris Noyvert, Andrew Beggs, Margaret Ng, Christina Halsey, Pamela Kearns, Paul Cheng, Francis Mussai

**Affiliations:** ^1^ Institute of Immunology and Immunotherapy, University of Birmingham Birmingham United Kingdom; ^2^ Institute of Cancer Sciences, Wolfson Wohl Cancer Research Centre, College of Medical, Veterinary and Life Sciences, University of Glasgow United Kingdom; ^3^ Institute of Cancer and Genomic Sciences, University of Birmingham Birmingham United Kingdom; ^4^ Department of Anatomic Pathology The Chinese University of Hong Kong Hong Kong; ^5^ Bio‐Cancer Treatment International Ltd Hong Kong

**Keywords:** arginine, ALL, arginase

## Abstract

Arginine is a semi‐essential amino acid that plays a key role in cell survival and proliferation in normal and malignant cells. BCT‐100, a pegylated (PEG) recombinant human arginase, can deplete arginine and starve malignant cells of the amino acid. Acute lymphoblastic leukemia (ALL) is the most common cancer of childhood, yet for patients with high risk or relapsed disease prognosis remains poor. We show that BCT‐100 is cytotoxic to ALL blasts from patients *in vitro* by necrosis, and is synergistic in combination with dexamethasone. Against ALL xenografts, BCT‐100 leads to a reduction in ALL engraftment and a prolongation of survival. ALL blasts express the arginine transporter CAT‐1, yet the majority of blasts are arginine auxotrophic due to deficiency in either argininosuccinate synthase (ASS) or ornithine transcarbamylase (OTC). Although endogenous upregulation or retroviral transduced increases in ASS or OTC may promote ALL survival under moderately low arginine conditions, expression of these enzymes cannot prevent BCT‐100 cytotoxicity at arginine depleting doses. RNA‐sequencing of ALL blasts and supporting stromal cells treated with BCT‐100 identifies a number of candidate pathways which are altered in the presence of arginine depletion. Therefore, BCT‐100 provides a new clinically relevant therapeutic approach to target arginine metabolism in ALL.

## Abstract 2

**What's new?**

While most children with acute lymphoblastic leukemia (ALL) are cured, only half of adults experience this outcome, and many patients suffer life‐long effects from the toxicities of chemotherapy. To overcome these challenges, therapies targeting novel mechanisms in ALL blast cells are needed. In our study, PEGylated recombinant human arginase (BCT‐100), which depletes the semi‐essential amino acid arginine, is shown to induce ALL cell death *in vitro*. In a xenograft model, BCT‐100 reduced disease burden and improved survival. Expression of the enzymes argininosuccinate synthetase and ornithine transcarbamylase, which synthesize recycle arginine intracellularly, are reduced in B‐cell ALL blasts, suggesting arginine auxotrophism.

## Introduction

Acute lymphoblastic leukemia (ALL) is the most common cancer of childhood. Significant progress has been made such that the majority of children will be cured of their disease through multi‐drug chemotherapy regimens. However, major challenges remain. For children who are diagnosed with high‐risk disease, or those who relapse the prognosis remains poor.^1^ Fewer than 50% of adults will be cured despite successful induction of a complete remission with chemotherapy.^2^ For those that are cured, the toxicities of treatment with chemotherapy over a 2‐ to 3‐year period remain a life‐long burden.^3^ Therefore, therapeutic strategies, which target ALL blasts through new mechanisms, but do not add to the cummulative toxicity, are urgently needed.

Arginine is a semi‐essential amino acid required for protein synthesis, cell division and a number of intracellular pathways that maintain cell survival.^4^, ^5^ Although whole body arginine levels are maintained through dietary intake and re‐synthesis, under conditions of high demand such as inflammation, pregnancy and cancer, arginine availability is limiting for on‐going cell growth and survival. Arginine is metabolized through the activity of Arginase I, II or iNOS enzymes. The enzymes ornithine transcarbamylase (OTC) and argininosuccinate synthase (ASS) provide the intracellular pathway in which normal cells can protect themselves by re‐synthesizing arginine from citrulline. However, cancer cells may be dependent on extracellular arginine for survival—arginine auxotrophism, due to the loss of ASS or OTC recycling enzyme expression; making them vulnerable to therapeutic arginine depletion.^6^

BCT‐100 is a clinical‐grade, PEGylated (PEG) recombinant human arginase that catalyses the conversion of arginine to ornithine and urea, leading to arginine depletion.^7^ BCT‐100 has shown significant activity against solid tumors and acute myeloid leukemia both pre‐clinically and in clinical trials.^8^, ^9^ Here, we investigate the role of arginine metabolism in ALL and the activity of BCT‐100 as a clinically relevant therapeutic approach for ALL.

## Material and Methods

### ALL patient samples

Blood samples were obtained from 21 patients with newly diagnosed ALL, before the start of treatment, at the Birmingham Children's Hospital (Supporting Table 1). Plasma was collected from samples taken at the time of diagnosis, before any therapy. Leukemic blasts were sorted from fresh peripheral blood mononuclear cells (following lymphoprep enrichment of whole blood) by CD19+ MACS beads, using Miltenyi LS columns. ALL samples were investigated within 24 hrs of blood sampling from patients. Bone marrow samples from 34 newly diagnosed adult and paediatric ALL patients were obtained from the Chinese Hospital, Hong Kong. Cerebrospinal fluid (CSF) samples from 20 ALL patients (diagnosis and 1 year into treatment) and 23 healthy controls at diagnosis were obtained.

### Cytotoxicity assay

Cell lines or sorted ALL blasts from patients were re‐suspended in RPMI‐1640 (Sigma), 10% heat‐inactivated arginine free fetal bovine serum (Sigma), glutamine (1X) (Sigma), penicillin–streptomycin (1X) (Sigma) and sodium pyruvate (1X) (Sigma). Approximately 2 × 10^5^ ALL blasts or 0.5 × 10^5^ cell lines were added to each well of 96‐well plates (Corning Costar). On day 1, BCT‐100 was added at final concentrations of 0, 200, 400, 600, 800, 1,000, 1,500, 2,000, 4,000 or 9,600 ng/mL to duplicate wells. The cytotoxicity of dexamethasone (600 ng/mL) was also tested in combination with BCT‐100. Cells were incubated for a further 72 hrs. The effect of arginine deprivation was similarly tested by culturing ALL cell lines and patients' blasts in SILAC arginine free RPMI‐1640 (Fisher Scientific), 10% heat‐inactivated arginine free fetal bovine serum (Fisher Scientific), glutamine (1X) (Sigma), penicillin–streptomycin (1X) (Sigma) and sodium pyruvate (1X) (Sigma).

### ALL murine xenografts

Non‐obese diabetic (NOD)/Shi‐scid/interleukin (IL)‐2 R severe combined immunodeficient γnull (NOG) mice aged 10–14 weeks were injected with 5 × 10^6^ REH‐green fluorescent protein (GFP) leukemia cells (ATCC). To assess the effect of BCT‐100 on engraftment, 20 mg/kg BCT‐100 was injected intravenously (i.v.) twice a week or after 1 week from engraftment. Bone marrow was harvested from the leg bones of mice killed after 4 weeks of treatment. To investigate the activity of BCT‐100 against patient‐derived ALL blasts *in vivo*, NOG mice were engrafted with 1 × 10^6^ human leukemia blasts sorted from the blood of a newly diagnosed patient with ALL.

Bone marrow engraftment was confirmed after 12 weeks by killing a sample population of mice and staining with anti‐human CD45 (% of CD45 on average 20%). A 20‐mg/kg BCT‐100 was injected twice a week. The frequency of human blasts (anti‐human CD45) was analyzed in bone marrow at the day of the mice were killed (15% weight lost).

For central nervous system (CNS) leukemia modeling, 6‐week‐old JAX NOD.Cg‐PrkdcscidIl2rgtm1Wjl/SzJ (NSG) (Charles River, Europe) mice were injected with 2 × 10^6^ REH cells *via* the tail vein. From day 14, the mice were treated with weekly i.v. 20 mg/kg BCT‐100. The mice were killed at day 33 when there was evidence of symptomatic leukemia with weight loss and early hind‐limb paralysis.

### Histology and immunohistochemistry

Paraffin‐embedded tissue sections of bone marrow trephines from 34 ALL patients at diagnosis were deparaffinised and rehydrated. Antigen retrieval was performed in 50 mM Tris/2 mM EDTA pH 9.0 using a Philips Whirlpool Sixth Sense microwave on a steaming program. Staining with anti‐human ASS (Abcam) and anti‐human OTC (Abcam) using the Novolink Polymer Detection System (RE7280‐K, Leica). Primary antibody incubation was performed overnight in a cold room. Sections were counterstained with Gill Nr 3 hematoxylin (Sigma Aldrich) and mounted in Aquatex (Merck).

CNS histology was prepared as described previously.^12^ Briefly, mouse heads were stripped of soft tissues, fixed in 10% neutral buffered formalin, decalcified and stained with hematoxylin and eosin. Images were captured using Hamamatsu NanoZoomer NDP scanner, and the area of CNS involvement quantified using HALO v2.0.1061.3 software (Indica Labs Inc.).

### RNA sequencing

RNA was derived from ALL blasts (REH cell line or human) sorted from the bone marrow of the murine xenografts described above, by CD19+ MACS bead selection, using Miltenyi columns. RNA was also derived from the CD19‐stromal fraction. Purity was checked by flow cytometry. Samples were prepared with the Illumina TruSeq RNA Sample Preparation Kit v2. They were sequenced on the Illumina HiSeq2000 platform using TruSeq v3 chemistry, over 76 cycles. Reads were mapped to hg19 (human) or mm10 (murine) genomes using STAR RNA‐Seq aligner software, version 2.5.1b.^13^ Number of reads per gene was counted by the same software. Read counts were normalized and the regularized fold change following treatment with BCT‐100 was calculated using DESeq2 package.^14^ Genes were ranked by the strength of the regularized log fold change.

### Mouse CSF experiments

CSF was collected from mice under terminal anesthesia with pentobarbital. A 25‐gauge needle was percutaneously inserted into the cisterna magna and CSF collected by gravity. Blood was collected immediately post‐mortem. Both CSF and blood were centrifuged at 2,000*g* for 15 min at 4°C. CSF supernatant and plasma were snap‐frozen and stored at −80°C until analysis.

### Mass spectroscopy

Aliquots of 1 μl of CSF or plasma were diluted 1:50 in a 20% water/50% methanol/30% acetonitrile solution and thoroughly mixed. The solution was spun at 16,000*g* for 10 min at 4°C, and the supernatant collected and analyzed by high‐performance liquid chromatography (HPLC)‐mass spectrometry. HPLC separation was achieved *via* a ZIC‐pHILIC column (SeQuant) with a Guard column (Hichrom), and metabolite mass/charge ratio measured with qExactive Orbitrap Mass Spectrometer after electrospray ionization (Thermo Scientific) operating in polarity switching mode.

### Statistical analysis

A Wilcoxon rank‐sum test was used to determine the statistical significance of the difference in unpaired observations between two groups (GraphpPad Prism). Correlations between parameters were evaluated using Spearman rank correlation analyses. *p* values are two‐tailed and where values were <0.05, they were considered statistically significant. For combination studies of BCT‐100 with dexamethasone, the interaction effect of the two drugs was tested in a two‐way analysis of variance (ANOVA).^10^ Analysis of synergism was assessed according to the Chou and Talalay method, using CompuSyn software (ComboSyn, NJ).^11^ ALL blasts from patients were cultured with BCT‐100 alone (0, 200, 400, 600, 800, 1,000, 1,200 and 2,400 ng/mL), dexamethasone (0, 200, 400, 600, 800, 1,000, 1,200 and 2,400 ng/mL) or both for 72 hrs. The percentage of viable cells relative to control after 72 hrs was measured by flow cytometry. Using this method, a CI at IC_50_ for individual patient samples is calculated, synergism is defined as CI < 1, while antagonism is CI > 1, and an additive effect is considered as CI = 1. For CNS histology quantification and metabolite abundance, results were analyzed by two‐tailed unpaired student's *t*‐tests.

### Study approval

In accordance with the Declaration of Helsinki, patient samples were obtained after written, informed consent before inclusion in the study. Regional Ethics Committee (REC Number 10/H0501/39) and local hospital trust research approval for the study was granted for United Kingdom hospitals and at the Chinese University Hospital, Hong Kong. The Birmingham Biomedical Ethics Review Subcommittee (BERSC) or the University of Glasgow Animal Welfare and Ethical Review Board (AWERB) approved all animal protocols in our study. Procedures were carried out in accordance with UK Home Office Guidelines and under Home Office License 60/4512.

## Results

### BCT‐100 arginine depletion reduced disease burden and prolongs survival in ALL murine xenografts

ALL remains the paradigm for metabolic therapies in the treatment of cancer, through the established use of PEG‐asparaginase‐ and methotrexate‐based chemotherapy regimens. Although acute myeloid leukemia has been shown to be dependent on arginine, the requirement of this amino acid in pre‐B ALL survival and proliferation is unclear. BCT‐100 lowers arginine levels *in vitro* and *in vivo* to undetectable levels.^9^ Screening of 3 B‐ALL cell lines REH, TOM1 and NALM6 revealed similar cytotoxicity profiles *in vitro* to BCT‐100 with 50% inhibitory concentration (IC_50_)s of 17.5–140 ng/mL. Less than 15% residual viable cells are present at concentrations of BCT‐100 >600 ng/mL, when arginine concentrations are undetectable in the supernatants (Supporting Information Fig. S1a). 2T‐ALL cell lines JURKAT and MOLT‐4 were similarly tested *in vitro*, revealing IC_50_ of 400 ng/mL and 150 ng/mL, respectively. JURKAT cells were more resistant to BCT‐100 *in vitro*, although at higher BCT‐100 doses <10% of residual viable cells remained (Supporting Information Fig. S1*b*).

**Figure 1:**
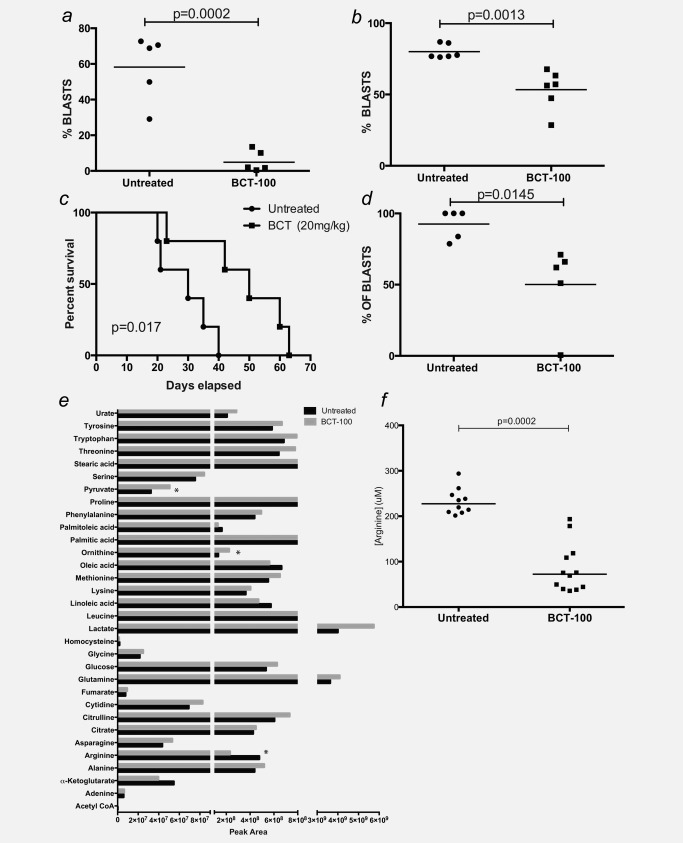
BCT‐100 arginine depletion decreases ALL disease burden *in vivo*. (*a*) NOG mice were injected with REH‐GFP ALL blasts. BCT‐100 (20 mg/kg) was given by i.v. injection twice a week from D+1. Bone marrow was sampled from the femurs after 2 weeks to assess hCD45+ cells by flow cytometry. BCT‐100 leads to significantly lower ALL engraftment. (*b*) NOG mice were injected with REH ALL blasts. BCT‐100 (20 mg/kg) was given by i.v. injection twice a week from D+14. Bone marrow was sampled from the femurs after 2 weeks to assess hCD45+ cells by flow cytometry. BCT‐100 leads to significantly lower ALL engraftment. Data are representative of two independent experiments. (*c*) NOG mice were injected with human ALL blasts, sorted from the blood of a newly diagnosed patient. BCT‐100 (20 mg/kg) was given by i.v. injection twice a week after engraftment was reached. Kaplan‐Meier curves showing a significant prolongation of survival in BCT‐100 treated mice. (*d*) NOG mice were injected with human ALL blasts, sorted from the blood of a newly diagnosed patient. BCT‐100 (20 mg/kg) was given by i.v. injection twice a week from D+1. Bone marrow was sampled from the femurs after 2 weeks to assess hCD45+ cells by flow cytometry. BCT‐100 leads to significantly lower ALL engraftment. (*e*) Levels of selected amino acids detected by HPLC‐MS in plasma of BCT‐100 treated or untreated NSG mice normalized to untreated mice. Data for arginine and immediate breakdown products are shown as individual graphs. (*f*) Plasma from control and BCT‐100 treated NOG mice was collected after 14 days. The concentration of arginine was determined by ELISA. BCT‐100 significantly lowers the plasma arginine concentration *in vivo*.

The effect of BCT‐100 was first tested against REH xenograft models of ALL, treated either early or late in leukemic engraftment. REH was selected as having representative *in vitro* cytotoxicity to the other cell lines and established *in vivo* engraftment kinetics. In both models, BCT‐100 treatment led to significant decrease in the burden of disease (early treatment (D + 1): 58.2% blasts in controls *vs*. 5.5% blasts in treated, *p* = 0.0002; Late treatment (D + 17): 80.1% blasts in controls *vs*. 53.3% blasts in treated, *p* = 0.0013) (Figs. 1 *a* and 1 *b*). To extend these findings, BCT‐100 was tested in a patient‐derived xenograft model of ALL. BCT‐100 led to a significant prolongation of murine survival (*p* = 0.017, Fig. 1 *c*) with an accompanying reduction in bone marrow disease (92.5% blasts in controls *vs*. 50.1% blasts in treated; *p* = 0.0145, Fig. 1 *d*). No blasts were identified in the blood or spleens of mice in either model during treatment, by flow cytometry, ruling out that decreases in bone marrow blast numbers was due to egress of these cells from the bone marrow (Supporting Information Fig. S1*c*).

Consistent with the specific catabolism by Arginase I, BCT‐100 induced a significant reduction in plasma arginine concentration (*p* = 0.0002) (Figs. 1 *e* and 1 *f*). Mass spectroscopy analysis of the plasma revealed a significant increase in ornithine (*p* = 0.0028), lactate (*p* = 0.001) and pyruvate levels (*p* = 0.015), with no other metabolic changes (Fig. 1 *e*). No evidence of toxicity or weight loss was seen in the BCT‐100 treated mice (Supporting Information Fig. S1*d*).

**Figure 2:**
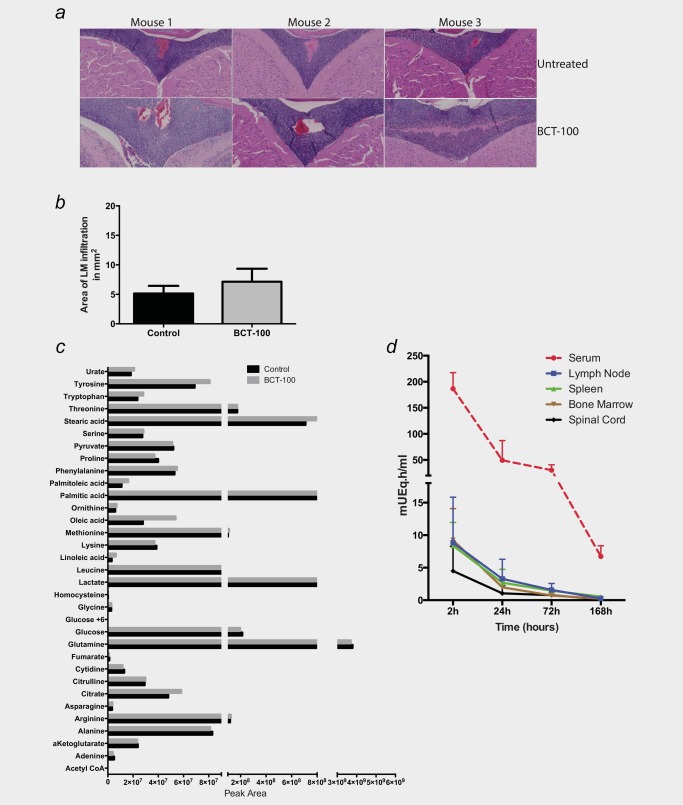
BCT‐100 does not affect CNS metabolic profile or ALL disease burden *in vivo*. (*a*) NSG mice were injected i.v. with REH cells and BCT‐100 (20 mg/kg) was given by i.v. injection weekly from D+14. Histology of treated and control mice showing no change in ALL engraftment in the CNS at the end of the experiment. Representative images from three of seven mice per group are shown. (*b*) Quantification of area of ALL CNS infiltration from xenografts showing no significant difference between control and treated mice. (*c*) Mass‐spectroscopy profiling of CSF from BCT‐100 and control mice revealing no significant change in metabolites. *p* values are all >0.05 (not shown). (*d*) Distribution of total radioactivity mice i.v. injected with ^125^I‐PEG‐BCT‐100. The concentration of total radioactivity in tissues of mice at 2, 24, 72 and 168 hrs after intravenous injection of ^125^I‐ PEG‐BCT‐100.The highest total radioactivity level was found in serum, followed by lymph node, spleen, bone marrow and spinal cord (*n* = 6 mice per time point).

At disease presentation, patients may present with blasts in the CSF. Using a xenograft model with established CSF ALL kinetics, we identified that BCT‐100 administered i.v. does not impact on CNS ALL disease (Figs. 2 *a* and 2 *b*), and CSF arginine (and other amino acid) concentrations are unchanged (Fig. 2 *c*).^12^ The result can be explained by the measurement of radiolabeled drug penetration into xenografts tissues. High concentrations of BCT‐100 were found in the blood (AUC: 6474.4 mUEq h/mL) with moderate levels in the key lymphoid compartments of the lymph node, spleen and bone marrow (Fig. 2 *d*, total; Supporting Information Fig. S1*e*). However, bioavailability of BCT‐100 was significantly lower in the spinal cord, supporting our findings above.

### ALL blasts are auxotrophic for arginine due to deficiencies in ASS or OTC expression

**Figure 3:**
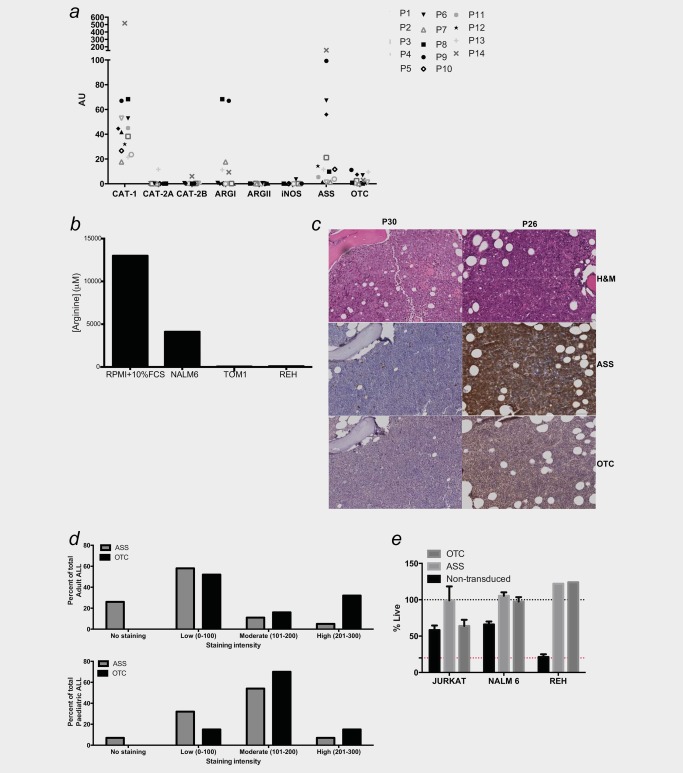
ALL blasts from patients are auxotrophic for arginine. (*a*) Arginine pathway component expression was determined by qPCR of sorted CD19+ ALL blasts from patients at diagnosis. Patients are identified by unique symbols, which are used consistently throughout the manuscript. AU: arbitrary units. (*b*) ALL cell lines significantly deplete arginine from the microenvironment. All data are representative of three independent experiments. (*c*) Staining of bone marrow samples from ALL patients at diagnosis with hematoxylin eosin (upper panel), anti‐ASS (centre panel) and anti‐OTC (lower panel) (Scale = 100 μm). Representative marrows from 2 of 35 patients showing positive antigen staining (right) and negative antigen staining (left). (*d*) Histoscores of ASS and OTC staining in adult (upper) and pediatric (lower) ALL bone marrow samples. (*e*) JURKAT, NALM6 and REH cell lines transduced with ASS or OTC genes have increased viability compared with wild‐type cell lines, in cultures with 150 ng/mL BCT‐100 (Histograms). At 600 ng/mL BCT‐100 cell viability of all lines was <15% (red dotted line). Representative of three experiments.

Arginine metabolism is based on three key cellular components—the transport of arginine from the extracellular microenvironment, catabolism of arginine into its products and the re‐synthesis of arginine from precursors. Arginine is transported from the microenvironment into cells principally by the Na+‐independent (System y+) family of transmembrane cationic amino acid transporters (CAT1, CAT2A and CAT2B). ALL blasts and non‐malignant B cells from healthy donors principally expressed CAT‐1, with an absence of CAT‐2A or CAT2B (Fig. 3 *a*; Supporting Information Fig. S1*f*). Proliferating blasts consumed arginine from the microenvironment (Fig. 3 *b*) and have a significant reduction in viability in the absence of arginine (Supporting Information Fig. S2*a*).

Once inside the cell arginine, is catabolized through the expression of Arginase I, Arginase II or NOS. Quantitative polymerase chain reaction (qPCR) of sorted patients' blasts (Fig. 3 *a*) and Western blot analysis of cell lines (Supporting Information Fig. S2b) showed that Arginase I is the main enzyme expressed. B cells from healthy donors similarly expressed Arginase I (Supporting Information Fig. S2c). Unlike in acute myeloid leukemia, where we found a significant reduction in plasma arginine, analysis of the plasma and CSF (Supporting Information Fig. S2d) of ALL patients revealed no significant reduction in arginine compared with healthy controls. There was no increase in arginase activity in cell culture supernatants or patient plasma (data not shown).

Cells may also source arginine from intracellular precursors through an arginine recycling pathway. The enzyme OTC converts ornithine into citrulline, which is converted to argininosuccinate through ASS expression. Argininosuccinate is finally converted back to arginine by argininosuccinate lyase (ASL). However, in cancer cells, OTC or ASS may be downregulated or absent, breaking the arginine biosynthesis pathway, making the cells dependent on extracellular arginine—arginine auxotrophism.^6^ B‐ALL cell lines expressed ASS and relatively lower expression of OTC (Supporting Information Fig. S2b), suggesting auxotrophism. No expression of ASS or OTC could be detected in normal B cells (Supporting Information Fig. S2c).

To investigate whether ALL blasts from patients have an arginine auxotrophic signature, 34 diagnostic bone marrow aspirates from adults and children with ALL were examined (Fig. 3 *c*). Of 19 adult ALL samples, 26% had no staining, 58% showed low, 11% showed moderate and 5% showed high ASS expression; while 0% had no staining, 52% showed low, 16% showed moderate and 32% showed high OTC expression (Fig. 3 *d*, upper panel). Of 13 pediatric pre‐B ALL samples, 7% had no staining, 32% had low and 54% had moderate ASS expression and 7% had high expression; while 0% had no staining, 15% had low, 70% had moderate and 15% had high OTC expression (Fig. 3 *d*, bottom panel). Ten adult and one childhood sample had absent/low expression of both enzymes. The predominance of ASS expression over OTC expression was reconfirmed in a second cohort of 14 patients by qPCR (Fig. 3 *a*). Two pediatric T ALL samples were received of which ASS or OTC expression was low for 1 patient and moderate for another patient (Supporting Information Fig. S2e). Western blots of T‐ALL cell lines JURKAT and MOLT‐4 confirmed expression of ASS but an absence of OTC (Supporting Information Fig. S2f).Therefore, the majority of ALL blasts had absent or low expression of at least one of the key enzyme steps in the arginine recycling pathway, suggesting a reliance on extracellular arginine import for survival.

### Impact of ASS and OTC on ALL blast viability

To directly understand the impact of arginine recycling enzymes on ALL blast viability under low arginine conditions, human ASS and OTC genes were transfected into B‐ALL cell lines (NALM6 and REH) and a T‐ALL cell line (JURKAT). Following flow cytometric sorting Western blots confirmed an increased expression of ASS and OTC proteins, compared with wild‐type controls (Supporting Information Fig. S3a). Consistent with the role of these enzymes, ASS or OTC transduced ALL blasts had increased viability compared with wild‐type cell lines under moderately low arginine conditions (150 ng/mL BCT‐100) Fig. 3 *e*). However, at higher concentrations of BCT‐100 (<600 ng/mL BCT), when arginine is undetectable in culture supernatants, these transduced enzymes had no effect, with <15% viable cells remaining (red dotted line). These findings indicate that increased ASS and OTC can help maintain viability, when arginine availability in the microenvironment is sub‐optimal. However, under extreme depletion of arginine recycling, enzyme upregulation cannot compensate. These findings support that therapeutic doses of BCT‐100 have been demonstrated to have activity against ASS+ or OTC+ cells of solid cancers in preclinical and human trials.

### BCT‐100 has activity against patient‐derived ALL blasts and is synergistic with dexamethasone

**Figure 4:**
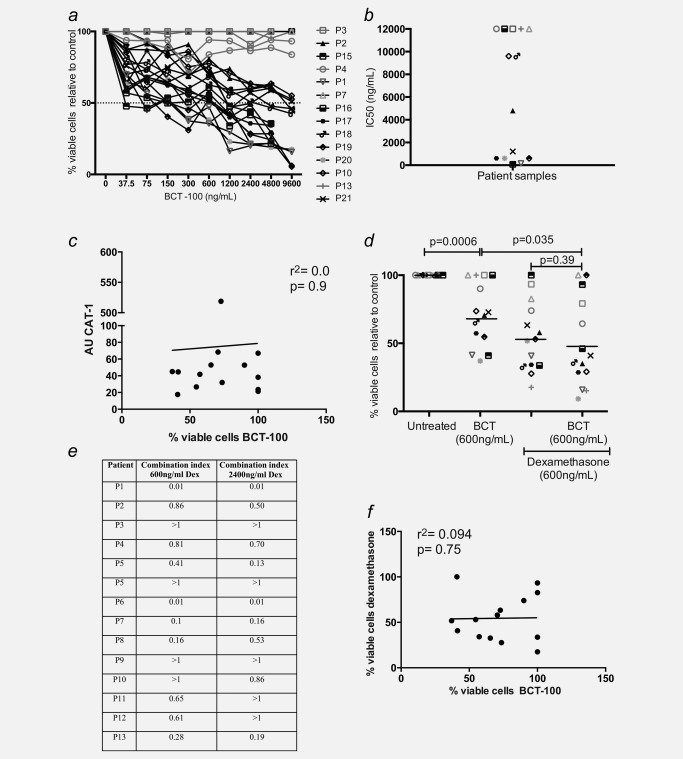
BCT‐100 is cytotoxic against ALL blasts from patients and synergises with dexamethasone. (*a*) ALL blasts from 14 newly diagnosed patients were cultured with BCT‐100 (0–9,600 ng/mL) for 72 hrs. The percentage of viable blasts relative to untreated was determined by flow cytometry. BCT‐100 leads to a dose‐dependent decrease in ALL blast viability. (*b*) IC_50_ values for the activity of BCT‐100 against ALL patient blasts are shown. (*c*) CAT‐1 expression does not correlate with the percentage of viable cells following 600 ng/mL BCT‐100. (*d*) ALL blasts from patients were cultured with 600 ng/mL BCT‐100 alone, 600 ng/mL dexamethasone or both for 72 hrs. The percentage of viable cells relative to control after 72 hrs was measured by flow cytometry. BCT‐100 cytotoxicity is synergistic in combination with dexamethasone (BCT *vs*. combination *p* = 0.035; dexamethasone *vs*. combination *p* = 0.39; two‐way ANOVA: *F*_(1,24)_ = 857.2, *p* < 0.0001). (*e*) Chou‐Talalay CI for individual patient samples showing synergy between BCT‐100 and dexamethasone. Synergism is defined as CI < 1. (*f*) The percentage of viable cells following treatment with 600 ng/mL BCT‐100 or 600 ng/mL dexamethasone was correlated. Sensitivity to BCT‐100 does not correlate with sensitivity to dexamethasone.

We described above that BCT‐100 can lower arginine, leading to reduced numbers of blasts in murine xenografts. To further investigate the effects of BCT‐100, ALL blasts were sorted from 14 fresh samples from patients at diagnosis, and treated with BCT‐100 (0–9,600 ng/mL) for 72 hrs. ALL blasts showed a range in sensitivity to BCT‐100 (Fig. 4 *a*) with IC_50_s from 75 to 9,600 ng/mL (Fig. 4 *b*). The majority of samples were sensitive to BCT‐100 with a significant reduction in viable ALL blasts. Five samples were completely resistant. No correlation with patients' clinical or genetic characteristics was seen (Supporting Information Table S1). No correlation with CAT‐1 protein expression was identified (Fig. 4 *c*). Indeed, blockade of CAT protein function with N‐nitro l‐arginine methyl ester (L‐NAME) had no effect on cell line viability (Supporting Information Fig. S3b).

### BCT‐100 induces necrotic cell death synergies with dexamethasone

Dexamethasone remains a cornerstone of ALL chemotherapy regimens at diagnosis and relapse.^16^ The combination of BCT‐100 with dexamethasone is significantly more cytotoxic than BCT‐100 alone (*F*_(1,24)_ = 857.2; *p* < 0.0001) (Fig. 4 *d*), and analysis of individual patient samples, showed that BCT‐100 synergized with dexamethasone (CI <1) for 10 of 14 samples (Fig. 4 *e*). BCT‐100 sensitivity did not correlate with sensitivity to dexamethasone (*r* = 0.094, *p* = 0.75), suggesting the two drugs have different mechanisms of activity and that dual treatment could be used in blasts resistant to dexamethasone (Fig. 4 *f*).

**Figure 5:**
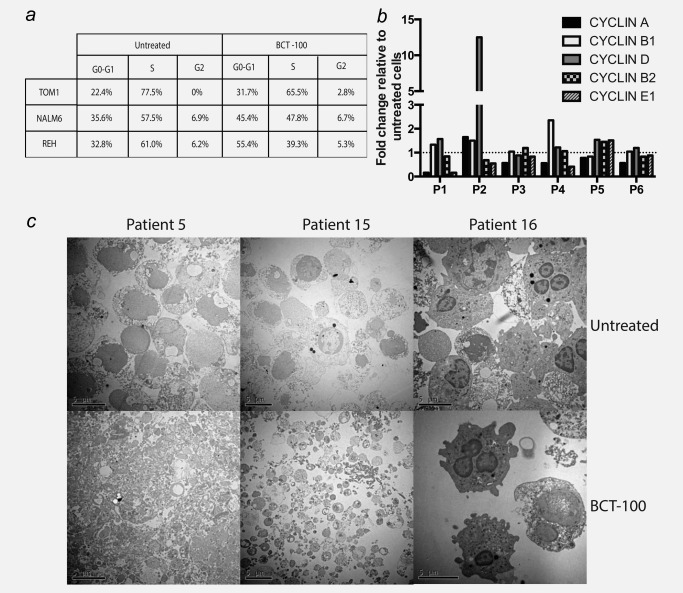
BCT‐100‐induced cell cycle arrest leads to necrotic cell death. (*a*) ALL cell lines were cultured with 600 ng/mL BCT‐100. Cell cycle analysis was performed after 72 hrs. BCT‐100 increases the percentage of cells in G0/G1 arrest. (*b*) Relative expression of cyclins A, B1, D, B2 and E1 in BCT‐100‐treated ALL patients' blasts compared with untreated controls (hashed line) were investigated by qPCR. Representative data of 6 patients are shown. (*c*) ALL blasts from patients were treated with BCT‐100 (600 ng/mL). Analysis of cell death was performed by transmission electron microscopy. Representative micrographs of three of six patients were shown. Upper panel: untreated cells. Lower panels: post treatment with 600 ng/mL BCT‐100. Features consistent with organelle enlargement, cell membrane permeablisation and cellular fragmentation with 600 ng/mL BCt‐100. Experiments performed on six separate occasions.

Arginine depletion has been shown to induce cell death through a number of different mechanisms, dependent on the cancer subtype.^9^, ^17^ Cell cycle analysis of ALL cell lines or patients' blasts showed an increase in cells in G0/G1 (Fig. 5 *a*) and increases in Cyclin B1 and D, compared with untreated cells (Fig. 5 *b*; Supporting Information Fig. S3c). BCT‐100 also led to an upregulation of CAT‐1, ASS and OTC in treated patients' blasts consistent with cellular attempts to compensate for low extracellular arginine (Supporting Information Figs. S3d and S3e). No significant differences in ASS, OTC or ARG1 expression were identified between sensitive and resistant samples (Supporting Information Fig. S3f).

Cell cycle arrest may lead to quiescence or cell death.^18^ BCT‐100 did not induce any significant increase in PI+/Annexin+ cells (Supporting Information Fig. S4a) or activation of caspases‐9 and −3 or PARP cleavage (Supporting Information Fig. S4b), confirming that apoptosis is not the mechanism of cell death. Similarly, no evidence for increased conversion of LC3‐I to LC3‐II was identified, suggesting autophagy is not the driver of cell death (Supporting Information Fig. S4c). Western blotting of ALL cell lines and two patients confirms no evidence for RIP1K activation (Necroptosis), ruling out any major contribution for this form of cell death (Supporting Information Figs. S5a and S5b). Examination of ALL cells treated with BCT‐100 by electron microscopy confirmed a complete breakdown of cellular membranes and organelles most consistent with necrosis (Fig. 5 *c*), as has been previously reported in AML.^9^

Arginine is central to a number of cellular processes. To examine the global effects of arginine deprivation on ALL blasts *in vivo*, sorted REH cells and patient ALL blasts from the bone marrows of control and BCT‐100 treated mice were subjected to RNA‐sequencing. The top 10 genes with the highest fold‐change in gene expression are shown in Supporting Information Tables S2a (REH) and S2*b* (Patient xenograft). In REH, the top differentially expressed gene was the hemoglobin beta subunit. Other genes of interest included the ferritin heavy chain 1 pseudogene 16 (FTH1P16) and the Ferritin heavy polypeptide 1 (FTH1). Arginine conversion into nitric oxide has been shown to influence the iron signaling pathway in myeloid cells.^19^ In blasts from patient‐derived xenografts, the top differentially expressed gene was the immunoglobulin lambda variable chain (IGLV4–69). Changes in cell adhesion genes CEACAM6 and ITAG6 were also prevalent, which are known to correlate with minimal residual disease and prognosis in ALL patients.^20^, ^21^

We revalidated by qPCR that CEACAM6 and ITAG6 expression is decreased in resistant blasts (Supporting Information Fig. S4c), consistent with their known contribution to chemotherapy resistance in ALL.

Stromal cells have been shown to protect T‐ALL blasts from arginine deprivation *in vitro*, although the effects of arginine depletion on the stromal cells themselves have never been reported. Murine bone marrow, depleted from blasts was subject to RNA‐sequencing (Supporting Information Table S2*c*). Arginine depletion led to a downregulation of a number of genes associated with protein synthesis including ribosomal proteins (Rps6‐ps1 and Rps3a3) and eukaryotic translation elongation factor 1 alpha 1 pseudogene. The granulocytic marker Ly6G was also upregulated consistent with previous reports of increased immature granulocytes in the bone marrow of arginase‐treated mice.^22^

In summary, these results show that ALL blasts require arginine for survival and proliferation, but their auxotrophism makes them a target for BCT‐100 arginine‐depletion therapy.

## Discussion

Investigation of cancer metabolism is undergoing a renaissance, and studies of the changes in metabolic profiles of ALL before and after chemotherapy suggest blasts induce significant changes in amino acids and lipids in the microenvironment.^23^ Although concentrations of metabolites in body compartments can be evaluated, clinical translation of the findings has remained limited due to a lack of available molecules which can target these metabolic requirements. The paradigm of metabolic therapy in ALL is the use of Asparaginase, which takes advantage of the failure of ALL blasts to express Asparagine synthetase, making blasts dependent on exogenous sources of asparagine.^24^ Although methotrexate is another established approach to target leukemic cell metabolism (through dihydrofolate reductase inhibition), it is only recently that an alternative strategy, arginine depletion, has become viable.^25^

Arginine is a semi‐essential amino acid that is taken in from the diet and metabolized *via* tissue specific expression of Arginase I, ASS and OTC enzymes in the liver, intestine and kidney, respectively. At the cellular level, arginine is catabolized by Arginase I, II or NOS enzyme expression into molecules such as ornithine, a precursor for polyamines, and NO.^26^ Under limiting conditions, intracellular arginine concentrations are usually maintained through the recycling of arginine by the expression of ASS, OTC and ASL enzymes.^27^ However, we have shown that in adult solid cancers and in AML, the expression of these enzymes may be abnormal.^7^, ^9^ ASS expression had previously only been reported in three ALL patient samples.^27^ We identify here that, in the majority of 48 samples, pre‐B ALL cells have absent or low expression of at least one of these enzymes. Pediatric ALL blasts generally had higher expression of ASS or OTC compared with blasts from adult patients. Expression of low‐moderate ASS and OTC in our 2T‐ALL patient samples is consistent with findings in T‐ALL cell lines and suggests T‐ALL could also be a suitable target for arginase therapy.^28^ ASS and OTC contribute to blast survival at moderately low arginine conditions, but encouragingly for clinical development of this molecule, blast expression of ASS and OTC cannot fully protect the cells from the cytotoxicity of BCT‐100 when arginine is depleted to undetectable levels. The regulation of ASS, OTC and ASL expression in cancers, and the advantages it provides to the malignant cell are not well understood.^29^, ^30^ In melanoma and osteosarcoma cell lines, decreased ASS expression enhanced cell proliferation through enhancement of pyrimidine synthesis.^31^ ASS expression can also influence lipid metabolism through AMPK regulation in hepatic cells, suggesting multiple downstream effects of this enzyme in cellular metabolism.^32^ ASS gene expression can be modulated by promotor hypermethylation, which correlates with aggressive phenotypes of myxofibrosarcomas, although the role of epigenetic modulation in the setting of ALL is unknown. Less is known about OTC regulation in cancer. In normal liver, two isoforms (OTC‐t1 and OTC_t2) have been described, suggesting a role for post‐transcriptional control.^33^ In rat‐derived hepatomas, treatment with azacitidine induced OTC gene activation, suggesting epigenetic regulation may play a role.^34^ Studies of rare patients with congenital ASS and OTC deficiencies may shed further insight into the function and control of these enzymes.^35^

We have shown that PEG‐recombinant human Arginase (BCT‐100) can lower arginine levels *in vitro*, *in vivo* and in man to undetectable levels.^7^, ^9^ We confirmed here that this drug leads to a specific catabolism of arginine into its by products, including ornithine, that induces cell cycle arrest with differences in cyclin profiles, that progresses to necrosis in both AML and ALL blasts. Differences in cell cycle regulation and their abnormalities in malignant cell development from lymphoblastic and myeloid lineages may account for these findings or that arginine deprivation acts through different mechanisms in these two cell types. Ultimately, the majority of blasts die by necrosis. It is possible that arginine concentrations are reduced rapidly in both the extracellular and intracellular microenvironment and the pathways which might otherwise protect cells from death or cause a more programmed cellular destruction, such as induction of anti‐apoptotic proteins or initiation of autophagy, are not engaged. A previous report identified that mesenchymal stromal cells can protect T‐ALL cell lines from apoptosis induction by arginine depletion.^36^ Interestingly, we identified that BCT‐100 arginine depletion leads to significant increases in pyruvate and lactate concentrations in the serum, suggesting a global metabolic switch toward glucose metabolism. In the context of leukemia, T‐ALL blasts with a reduced glutamine dependence are suggested to undergo a metabolic switch toward glucose metabolism.^37^ Arginine supplementation can also induce indirect changes in glucose regulation *via* insulin‐induced phosphorylation of Akt in muscle and adipose tissue of diabetic rats.^38^ Thus, these glycolytic changes likely derive from non‐leukemic tissues. RNA‐sequencing of leukemic blasts after treatment with BCT‐100 revealed alterations in the iron metabolic process, including FTH1P16 and the FTH1. These findings are consistent with reports that nitric oxide can regulate gamma‐globulin, H‐ferritin and transferrin in AML cell lines or transfected fibroblasts.^39^ Stromal cells may compensate for arginine depletion by upregulating OTC, and contributing to leukemia resistance to this therapy. We found no evidence of significant alterations in the arginine pathway genes in either blasts or stromal cells by RNA‐sequencing, consistent with our previous report in AML blasts, and that of others, that pathways of resistance likely lie outside of arginine metabolism.^40^ Little is known about how ALL blasts remain resistant to metabolic therapies, as few are in current clinical practice.

Within the duration of our *in vivo* experiments, we saw no changes in CSF arginine or other amino acids, and no effect on CNS ALL disease. Although BCT‐100 has high bioavailability in the hematological compartments, penetration into the CNS was low. The status of the blood–brain barrier in leukemia patients is likely to be normal. We have previously shown that the ability of ALL blasts to enter the CSF is a generic mechanism.^12^ Similar molecules such as PEG‐Asparaginase do not enter the CSF but do lead to a depletion of asparagine from the CSF in children with ALL.^41^ Pegylated gene vectors have been reported to enter the brain in CNS tumor models, although here the blood–brain barrier may be abnormal and the molecules are much smaller.

BCT‐100 is undergoing clinical evaluation in a number of settings. A Phase I clinical trial of BCT‐100 has been completed in adults with relapsed/refractory hepatocellular carcinoma (HCC—NCT00988195) and the data describe an excellent safety profile and predictable pharmacokinetics and pharmacodynamics (PK/PDs).^42^ The trial demonstrated that, at 1600 U/kg BCT‐100 given i.v. over 1 hr at weekly intervals, plasma arginine falls below 8 μM (lowest detectable range) and is maintained for up to 166 hrs. Doses of BCT‐100 up to 2,500 U/kg were also administered with no maximum tolerated dose reached. A 1,600‐U/kg was chosen as the lowest effective dose (Optimal Biological Dose) to take forwards in adults. Toxicities were mainly NCI‐CTCAE grade 1 or 2—abdominal pain, diarrhea and transaminitis. Notably, the relapsed HCC population has significant transaminitis/hyperbilirubinemia due to disease location. A separate Phase I trial is underway in other adult solid malignancies (NCT02285101). One Phase II trial has been completed in 20 heavily pretreated adults with HCC (NCT01092091), which showed an improvement in overall survival in patients who achieved adequate arginine depletion.^8^ BCT‐100 was well tolerated, with a good toxicity profile similar to that reported in the Phase I study. BCT‐100 is currently under clinical development for adults with acute myeloid leukemia and children with relapsed/refractory solid and hematological cancers. The effect of BCT‐100 on CNS disease and arginine levels will be determined in these upcoming trials. Unlike alternative arginine depletion strategies, no evidence of immunogenicity or neutralizing antibodies which prevent arginine degradation have been reported for BCT‐100.^43^, ^44^, ^45^ Therefore, our study provides an exciting rationale for further clinical translation of BCT‐100 arginase‐based therapies for ALL and other malignancies.

## Supporting information

Supporting Information Figure 1

Supporting Information Figure 2

Supporting Information Figure 3

Supporting Information Figure 4

Supporting Information Figure 5

Supporting Information Figures

Supporting Information Table 1

Supporting Information Table 2

Supporting Information
