# Supplementary material for: The arginine metabolome in acute lymphoblastic leukemia can be targeted by the pegylated‐recombinant arginase I BCT‐100
Source: Int J Cancer. 2017 Dec 26;142(7):1490–502. doi: 10.1002/ijc.31170 (PMC5849425; doi:10.1002/ijc.31170)
Supplement: Supplementary file 6 — Supporting Information Figures [file IJC-142-1490-s006.docx]

**Supplementary Figure Legends:**

**S1: Effect of BCT-100 on ALL in vitro and in vivo**

a) TOM1, REH, and NALM6 cell lines were cultured with BCT-100 (0-2400ng/mL) for 72hours. The percentage of viable cells relative to untreated controls was determined by flow cytometry. BCT-100 leads to a dose-dependent decrease in ALL cell line viability b) JURKAT and MOLT4 cell lines were cultured with BCT-100 (0-2400ng/mL) for 72hours. The percentage of viable cells relative to untreated controls was determined by flow cytometry. BCT-100 leads to a dose-dependent decrease in ALL cell line viability c) Representative flow cytometry plots of the percentage of REH blasts in the blood, spleen or bone marrow of xenograft mice at the time of sacrifice d) Untreated and BCT-100 NOG mice engrafted with REH-GFP ALL showed no significant difference in body weight in response to treatment e) Distribution of TCA-precipitable radioactivity in mice intravenously injected with ^125^I- PEG-BCT-100. The concentration of total radioactivity in tissues of mice at 2, 24, 72 and 168 hours after intravenous injection of ^125^I- PEG-BCT-100, confirms radioactivity measured is due to labelled BCT-100 and not small molecule degradants. The highest radioactivity level was found in serum, followed by lymph node, spleen, bone marrow, and spinal cord, as before. (n=6 mice per timepoint). f) CAT-1, CAT-2A and CAT-2B expression was determined by qPCR of sorted CD19+ B lymphocytes from 5 healthy donors

**S2: Arginine auxotrophic profile of ALL blasts**

a) Cell lines or patients’ cells were cultured in normal or arginine free-media for 72hours. The percentage of viable cells relative to untreated controls was determined by flow cytometry. Arginine free conditions lead to a loss of ALL cell line viability b) Expression of Arginase I, Arginase II, iNOS, ASS, and OTC in whole cell lysates of B ALL cell lines was determined by immunoblotting. Actin was used as the housekeeping gene to ensure equal loading c) Expression of Arginase I, Arginase II, iNOS, ASS, and OTC in whole cell lysates of healthy donor CD19+ B cells was determined by immunoblotting. Actin was used as the housekeeping gene to ensure equal loading d) Plasma and CSF from leukemia patients were analysed for the level of arginine by ELISA. Healthy donors were used has control. e) Histoscores of ASS and OTC staining in 2 T-ALL bone marrow samples form patients. f) Expression of Arginase I, Arginase II, iNOS, ASS, and OTC in whole cell lysates of T ALL cell lines was determined by immunoblotting. Actin was used as the housekeeping gene to ensure equal loading

**S3: Intracellular changes in ALL blasts following BCT-100 treatment**

a) Increased expression of ASS or OTC protein in B-ALL or T ALL cell lines, compared to mock gene transduction, following retroviral transduction of the enzymes. Representative plots shown. b) Cell lines were cultured with L-NAME (0-500mM) for 72hours. The percentage of viable cells relative to untreated controls was determined by flow cytometry. L-NAME led to no significant change in ALL cell line viability. c) Relative expression of cyclins A, B1, D, B2 and E1 in BCT-100 treated ALL cell lines compared to untreated controls (hashed line) were investigated by qPCR. Representative data of 3 individual experiments d) Relative expression of CAT-1 in BCT-100 treated ALL blasts from 6 patients compared to untreated controls (hashed line) were investigated by qPCR e) Relative expression of ASS, OTC, ARGI, ARG II and iNOS in BCT-100 treated ALL blasts from 6 patients compared to untreated controls (hashed line) were investigated by qPCR f) Expression of ASS, OTC, and ARGI in sensitive versus resistant blasts.

**S4: BCT-100 does not activate apoptotic or autophagy pathways in ALL blasts**

a) ALL cell lines were treated with BCT-100 for 72hours and stained with Annexin-PI. Percentages of Annexin and PI positive cells, compared to untreated controls, is shown, as tested by flow cytometry. No significant increase in Annexin+PI+ cells is seen, confirming the lack of apoptosis. Representative of 3 independent experiments b) Cell lines were cultured alone (A), PEG (B), BCT-100 600ng/ml (C) or BCT-100 2400ng/ml (D). Expression of PARP, Caspase 9, and Caspase 3 in whole cell lysates was determined by immunoblotting. Actin was used as the housekeeping gene to ensure equal loading. No significant cleavage of PARP, Caspase 9 or 3 is seen, indicating the apoptosis pathway was not activated c) ALL cell lines were treated with BCT-100 in the presence or absence of bafilomycin. Whole cell lysates were tested for LC3-I and LC3-II turnover by immunoblotting at 0H,24H,48H, 72H of culture. No increase in conversion of LC3-I to LC3-II is seen, confirming the lack of autophagy induction above baseline

**S5: BCT-100 synergy with dexamethasone does not involve apoptosis or necroptosis**

a) Cell lines were cultured alone (A), BCT-100 600ng/ml (B), Dexamethasone 600ng/ml (C) or BCT-100 with Dexamethasone (D). Expression of apoptosis and necroptosis pathway proteins in whole cell lystaes was determined by immunoblotting. Actin was used as the housekeeping gene to ensure equal loading. b) Patients’ blasts were cultured alone (A), BCT-100 600ng/ml (B), Dexamethasone 600ng/ml (C) or BCT-100 with Dexamethasone (D). Expression of apoptosis and necroptosis pathway proteins in whole cell lystaes was determined by immunoblotting. Actin was used as the housekeeping gene to ensure equal loading. c) Relative expression of ITAG6 and CD66c in ALL blasts from BCT-100 treated mice compared to untreated control was investigated by qPCR. Representative data of 3 individual experiments.
