# Supplementary material for: The arginine metabolome in acute lymphoblastic leukemia can be targeted by the pegylated‐recombinant arginase I BCT‐100
Source: Int J Cancer. 2017 Dec 26;142(7):1490–502. doi: 10.1002/ijc.31170 (PMC5849425; doi:10.1002/ijc.31170)
Supplement: Supplementary file 7 — Supporting Information Table 1 [file IJC-142-1490-s007.docx]

Supplementary Table 1: Table of patient clinical characteristics

| Patient ID | Time point | Age (years) | Sex | Peripheral Blast count at diagnosis (x10^9^/L) | Cytogenetics/ Karyotype |
| --- | --- | --- | --- | --- | --- |
| P1 | Diagnosis | 12 | M | 2.5 | High hyperdiploidy |
| P2 | Diagnosis | 9 | F | 4 | t(12;21)(p13;q22) |
| P3 | Diagnosis | 4 | F | 25.4 | High hyperdiploidy |
| P4 | Diagnosis | 3 | F | 15.2 | Normal karyotype |
| P5 | Diagnosis | 10 | F | 28.7 | VH1-JHC and VH5-JHC rearrangements; Loss of 9p. |
| P6 | Diagnosis | 4 | M | 0.2 | High hyperdiploidy |
| P7 | Diagnosis | 6 | M | 46.8 | Del(6q) (T-ALL) |
| P8 | Diagnosis | 2 | M | 4.3 | Normal karyotype |
| P9 | Diagnosis | 14 | M | pancytopenia | Normal karyotype |
| P10 | Diagnosis | 4 | M | 11.6 | Variant IgH gene rearrangement |
| P11 | Diagnosis | 4 | F | 17 | Normal karyotype |
| P12 | Diagnosis | 3 | M | 7.2 | Gain of one copy of CEPX, CEP6, CEP7, CEP10, CEP17, RUNX1 |
| P13 | Diagnosis | 10 | M | 0.8 | t(12;21)(p13;q22) |
| P14 | Diagnosis | 14 | F | 292.2 | T(X;14) (IGH-CRLF2) |
| P15 | Diagnosis | 5 | M | 6.5 | High hyperdiploidy |
| P16 | Diagnosis | 9 | M | 4.9 | Gain of copy of IgH, normal karyotype |
| P17 | Diagnosis | 3 | F | 3.6 | VH3-JHc rearrangement, normal karyotype |
| P18 | Diagnosis | 6 | F | 1.9 | t(10;11)(p12;q24) (T-ALL) |
| P19 | Diagnosis | 5 | M | 15.3 | Normal karotype |
| P20 | Diagnosis | 2 | F | 2.4 | High hyperdiploidy |
