# Supplementary material for: The arginine metabolome in acute lymphoblastic leukemia can be targeted by the pegylated‐recombinant arginase I BCT‐100
Source: Int J Cancer. 2017 Dec 26;142(7):1490–502. doi: 10.1002/ijc.31170 (PMC5849425; doi:10.1002/ijc.31170)
Supplement: Supplementary file 8 — Supporting Information Table 2 [file IJC-142-1490-s008.docx]

**Supplementary Table 2a: Table of ranked gene expression in arginase control vs. treated REH cells as determined via RNA-seq**

| Rank | Gene | Ensembl ID | Gene description | Regulated Log2(fold change) |
| --- | --- | --- | --- | --- |
| 1 | HBB | ENSG00000244734 | Hemoglobin subunit beta | 0.953 |
| 2 | TUBA4A | ENSG00000127824 | Tubulin alpha 4a | 0.602 |
| 3 | FTH1P16 | ENSG00000227376 | Ferritin heavy chain 1 pseudogene 16 | 0.56 |
| 4 | ACTBP11 | ENSG00000188460 | Actin, beta pseudogene 11 | 0.556 |
| 5 | HSPB1 | ENSG00000106211 | Heat shock protein family B (small) member 1 | -0.547 |
| 6 | MALAT1 | ENSG00000251562 | Metastasis associated lung adenocarcinoma transcript 1 | -0.51 |
| 7 | TMSB4XP8 | ENSG00000187653 | Thymosisn beta 4, X-linked pseudeogene 8 | 0.495 |
| 8 | FTH1 | ENSG00000167996 | Ferritin heavy polypetide 1 | 0.495 |
| 9 | UCP2 | ENSG00000175567 | Uncoupling protein 2 | 0.469 |
| 10 | THBS1 | ENSG00000137801 | Thrombospondin 1 | 0.432 |

**Supplementary Table 2b: Table of ranked gene expression in arginase control vs. treated patient ALL cells as determined via RNA-seq**

| Rank | Gene | Ensembl ID | Gene description | Regulated Log2(fold change) |
| --- | --- | --- | --- | --- |
| 1 | IGLV4-69 | ENSG00000211637 | Immunoglobulin lambda variable | 1.075 |
| 2 | AC064834.1 | ENSG00000224099 | Linc RNA | -0.84 |
| 3 | CEACAM6 | ENSG00000086548 | Carcinoembryonic antigen related cell adhesion molecule 6 | -0.838 |
| 4 | ITGA6 | ENSG00000091409 | Integrin subunit alpha 6 | -0.804 |
| 5 | NRP1 | ENSG00000099250 | Neuropilin 1 | -0.78 |
| 6 | GPRIN3 | ENSG00000185477 | GPRIN family member 3 | -0.776 |
| 7 | CDC42EP3 | ENSG00000163171 | CDC42 effector protein 3 | -0.696 |
| 8 | NPY | ENSG00000122585 | Neuropeptide Y | -0.693 |
| 9 | MTATP6P1 | ENSG00000248527 | Mitochondrially encoded ATP synthase 6 | 0.617 |
| 10 | MT-ATP6 | ENSG00000198899 | Mitochondrially encoded ATP synthase 6 | 0.61 |

**Supplementary Table 2c: Table of ranked gene expression in arginase control vs. treated murine stromal cells as determined via RNA-seq**

| Rank | Gene | Ensembl ID | Gene description | Regulated Log2(fold change) |
| --- | --- | --- | --- | --- |
| 1 | Rps6-ps1 | ENSMUSG00000063875 | Ribosomal protein S6, pseudogene 1 | -0.744 |
| 2 | Rps3a3 | ENSMUSG00000059751 | Ribosomal protein S3A3 | -0.726 |
| 3 | Gm6170 | ENSMUSG00000100078 | Eukaryotic Translation Elongation Factor 1 alpha 1 pseudogene | -0.714 |
| 4 | BC100530 | ENSMUSG00000071561 | cDNA sequence BC100530 | 0.71 |
| 5 | Ly6g | ENSMUSG00000022582 | Lymphocyte antigen 6 complex, locus G | 0.672 |
| 6 | Stfa2 | ENSMUSG00000022902 | Stefin A2 | 0.64 |
| 7 | Asprv1 | ENSMUSG00000033508 | Aspartic peptidase, retroviral-like 1 | 0.633 |
| 8 | Gm13456 | ENSMUSG00000082536 | Eukaryotic Translation Elongation Factor 1 alpha 1 pseudogene | -0.631 |
| 9 | Cd52 | ENSMUSG00000000682 | CD52 antigen | 0.608 |
| 10 | Gm15796 | ENSMUSG00000089646 | Predicted gene 15796 | -0.584 |
