# Supplementary material for: The arginine metabolome in acute lymphoblastic leukemia can be targeted by the pegylated‐recombinant arginase I BCT‐100
Source: Int J Cancer. 2017 Dec 26;142(7):1490–502. doi: 10.1002/ijc.31170 (PMC5849425; doi:10.1002/ijc.31170)
Supplement: Supplementary file 9 — Supporting Information [file IJC-142-1490-s009.docx]

**Supplemental Methods**

*BCT-100*

Clinical-grade BCT-100 was gifted by Bio-Cancer Treatment International, Hong Kong. Briefly BCT-100 was made by expressing human Arginase I in an E.Coli system, purified (>98% purity) and then pegylated with mPEG-SPA5000 under GMP conditions.^7^

*Cell lines*

REH, TOM1, NALM6, MOLT4, JURKAT (ATCC) cell lines were cultured in RPMI-1640 (Invitrogen, CA, USA) with 10% heat-inactivated fetal bovine serum, glutamine (1x), sodium pyruvate (1x) and Penicillin-Streptomycin (RPMI 10%) using T-75 flasks kept in a humidified air atmosphere with 5% CO_2_ at 37^o^C.N_w_-Nitro-L-arginine methyl ester hydrochloride (L-NAME) (Sigma) was added to culture media, where indicated at concentrations from 0-500mM, to block CAT function.

*Arginine ELISA*

The concentration of arginine in media, human plasma or CSF was quantified using a competitive enzyme linked immunoassay (Immunodiagnostik K7733) according to the manufacturers’ instructions. In brief this assay uses a competitive enzyme immunoassay in which L-arginine is derivatized from samples, and competes with an L-arginine-tracer for binding of polyclonal antibodies, in the microtiter wells. The concentration of the tracer-bound antibody is inversely proportional to the L-arginine concentration in the samples. Plasma collected from the blood of NOG ALL murine xenografts was similarly tested.

*RT-Q-PCR analysis*

RT-Q-PCR was used to detect Arginase I, Arginase II, iNOS, CAT-1, CAT-2A, CAT-2B, ASS and OTC in patient-derived ALL blasts and B-lymphocytes. RNA was extracted using an RNeasy Mini kit (Qiagen). cDNA was prepared using SuperScriptTM III Reverse Transcriptase (Invitrogen) following the manufacturer’s instructions. RT-Q-PCR was done in duplicate using FAST SYBR Green Master Mix (Applied Biosystems) and the Applied Biosystems 7500 Fast Real-Time PCR system. Analysis of gene expression was calculated according to 2^-ΔT^ method described by Livak et al. plotted as arbitrary units of mRNA relative to GAPDH. Primer sequences (Eurofins) were:

| Cyclin A | 5'-AATGGGCAGTACAGGAGGAC-3’ | Forward |
| --- | --- | --- |
|  | 5'-CCACAGTCAGGGAGTGCTTT-3' | Reverse |
| Cyclin B1 | 5'-CATGGTGCACTTTCCTCCTT-3' | Forward |
|  | 5'-AGGTAATGTTGTAGAGTTGGTGTCC-3' | Reverse |
| Cyclin E | 5'-GGCCAAAATCGACAGGAC-3' | Forward |
|  | 5'-GGGTCTGCACAGACTGCAT-3' | Reverse |
| Cyclin D | 5'-GAAGATCGTCGCCACCTG-3’ | Forward |
|  | 5'-GACCTCCTCCTCGCACTTCT-3’ | Reverse |
| Cyclin B2 | 5'-GCGTTGGCATTATGGATCG-3’ | Forward |
|  | 5'-TCTTCCGGGAAACTGGCTG-3’ | Reverse |
| Arginase I | 5’-GTTTCTCAAGCAGACCAGCC-3’ | Forward |
|  | 5'-GCTCAAGTGCAGCAAAGAGA-3’ | Reverse |
| Arginase II | 5'-ATGTCCCTAAGGGGCAGCCTCTCGCGT-3’ | Forward |
|  | 5'-CACAGCTGTAGCCATCTGACACAGCTC-3’ | Reverse |
| iNOS | 5'-CCTCAAGCTATCGAATTTGTC-3’ | Forward |
|  | 5'-TTGCCATTGTTGGTGGAGTA-3’ | Reverse |
| ASS | 5'-GGGGTCCCTGTGAAGGTGACC-3’ | Forward |
|  | 5'-CGTTCATGCTCACCAGCTC-3’ | Reverse |
| OTC | 5'-AGTTATTTAACCAGCGTGTTT-3’ | Forward |
|  | 5'-TCCCCCTAAAGTGAATAAGTG-3’ | Reverse |
| CAT 1 | 5'-ATGGGTGGAAACGCTGATGATAC-3' | Forward |
|  | 5'-ACCTTGCCTGTTAAGTCTGGGTG-3' | Reverse |
| CAT 2A | 5'-TTAACACTTATGATGCCGTACTACCT-3' | Forward |
|  | 5'-GCAACTGGTGACTGCCTCTTACT-3' | Reverse |
| CAT 2B | 5'-ATGCCTCGTGTAATCTATGCTATG-3 ‘ | Forward |
|  | 5'-ACTGCACCCGATGATAAAGTAGC-3 | Reverse |
| GAPDH | 5'-CCAGCCGAGCCACATCGCTC-3’ | Forward |
|  | 5'-ATGAGCCCCAGCCTTCTC-3’ | Reverse |

*Immunohistochemistry scoring*

Antigen expression in immunohistochemistry sections were assigned independently by 2 experienced pathologists, as described by Nenutil et al.^46^ Briefly, to evaluate the immunostaining intensity each slide was examined on an Olympus BX51 microscope. Representative 400x magnification fields of at least 100 tumor cells were selected and photographed with an Olympus DP70 camera and accompanying image software. Fields were assigned an antigen staining intensity score of 0 = negative, 1= weak, 2 = moderate, 3 = strong. The product of the percent positive cells and staining intensity was then derived to create a histoscore of 0-300 for each high power field. A final histoscore was then given to each specimen for each antigen.

*Arginine depletion assay*

1x10^6^ cell lines were plated in 24 well plate (Corning Costar) in 1ml RPMI 10% for 48 hours. The supernatant were harvested and analysed for Arginine concentration by ELISA (Immunodiagnostik). RPMI 10 % has been used as control.

*Flow cytometric analysis*

Blasts from cell lines and patient samples were collected and labelled with propidium iodide (PI) to assess viability by flow cytometry. The relative percentage of viable cells at the end of the assay (72hours) was calculated using the following formula: (mean no. of viable blasts recovered in treatment wells/mean no. of viable blasts in untreated wells x 100). Apoptosis was estimated by cells being re-suspended in 1x Annexin Binding Buffer and labelled with PI and Annexin conjugated to phycocyanin (FITC) (FITC Annexin V Apoptosis Detection Kit I, BD Pharmingen). The cells were analysed with a Cyan flow analyser (Beckman Coulter) using FlowJo software (Tree Star Inc.). The 50% inhibitory concentration (IC_50_) is defined as the concentration of BCT-100 that killed 50% of the viable cells at the termination of the assay.

Cell cycle analysis was performed using PI staining and flow cytometry. 1x10^6^ cells/well incubated with RPMI 10% with or without BCT-100 (600 ng/mL) in 24 well plates for 72 hours were harvested, washed twice in PBS and fixed in cold ethanol for 1h at 4^o^C. Following washing with PBS, cells were stained with PI solution and 50µl of RNase A stock solution (10μg/mL, Invitrogen) at 4^o^C for 3 hours before analysis with a Cyan flow analyser in combination with ModFit software.

*Immunoblotting*

Following cell lysis (20nM Tris-Hcl pH7.5, 150nM NaCl, 2mM EDTA, 1.0% triton X-100 and protease and phosphatase inhibitors (Roche Applied Science, Indianapolis, IL) equal amounts of protein were loaded onto 12% Tris-Glycine SDS-PAGE (BioRad) gels and transferred to PVDF membranes. Hybridisation was carried out using antibodies to PARP, caspases -3, and -9, LC3 (Cell Signalling), ARGI, ARGII, iNOS and actin (Santa Cruz). HRP-conjugated secondary antibodies, goat anti-rabbit (Cell Signalling), and sheep anti-mouse (GE Healthcare) were used for blots, which were developed with ECL substrate (BioRad) and exposed on Kodak film.

*Transmission Electron microscopy*

ALL blasts were treated with BCT-100 (600ng/ml) in culture for 72hours. Following harvesting they were fixed in 2.5% glutaraldehyde followed by 1% osmium tetroxide. The samples were dehydrated through ethanol and embedded in propylene oxide/resin mixture at 60°C for 16h prior to sectioning at 80 nm in thickness and placement on 300 mesh copper slot grids for examination by transmission electron microscopy.

*Distribution of ^125^I-labelled PEG-BCT-100*

Pharmacokinetics of PEG-BCT-100 was studied with ^125^I-labelled PEG-BCT-100. A dose of 260 Units of ^125^I-labelled PEG-BCT-100 was given intravenously to murine xenografts. Mice were sacrificed at 2, 24, 72 and 168 hours after injection and organs harvested. Molecular exclusion HPLC and TCA precipitation method was used to study the distribution of PEG-BCT-100 in each mouse (n=6 mice per timepoint)*.*

*Retroviral Transduction*

Cell lines were retrovirally transduced with pMP71 ASS-GFP and OTC-GFP retroviral plasmids by spinfection as previously described.^47^ Briefly, Phoenix Ampho cells were transfected with 10µg of plasmid DNA using Fugene 6 transfection reagent [Promega]. Retrovirus was collected 48 hours later, added to retronectin-coated plates, and centrifuged at 2000 x g, 32°C for 2 hours. Cell lines to be transduced were harvested, added to retrovirus-coated plates at 1x10^6^ cells/ml, centrifuged at 500 x g for 5 minutes and incubated at 37°C, 5% CO_2_. GFP^+^ cells were FACS sorted 4-6 days later.
